# Supplementary material for: Immune correlates of anti-BCMA CAR-T products idecabtagene vicleucel and ciltacabtagene autoleucel in a real-world cohort of patients with multiple myeloma
Source: Nat Commun. 2025 Jul 4;16:6154. doi: 10.1038/s41467-025-60980-2 (PMC12229333; doi:10.1038/s41467-025-60980-2)
Supplement: Supplementary file 1 — Supplementary Information [file 41467_2025_60980_MOESM1_ESM.pdf]

Supplemental Figure 1

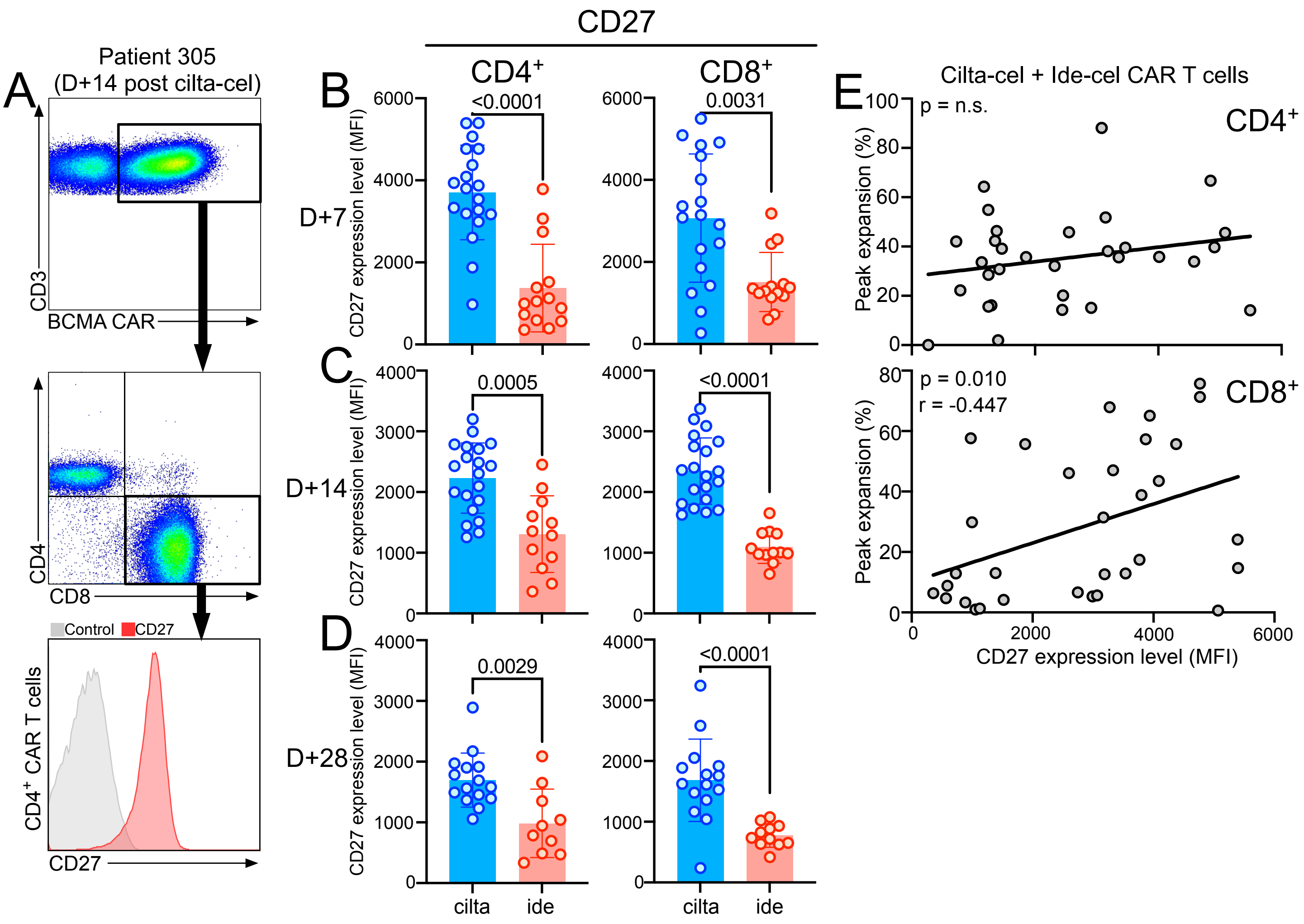

<u>Supplemental Figure 1:</u> Increased expression of CD27 on CAR T from myeloma patients receiving cilta-cel is associated with an enhanced expansion

(A) Dot plots and histogram on the left explain the gating strategy used to assess expression of surface markers such as CD27 on the CAR T cells. Within the histogram, the gray histogram represents an FMO control and the red histogram represents staining with the relevant monoclonal antibody. Bar graphs in the middle show surface expression levels of CD27 on CD4$^+$ or CD8$^+$ CAR T cells in the two groups of patients (blue = cilta-cel, red = ide-cel) on **(B)** D+7, **(C)** D+14, and **(D)** D+28. Individual values indicate mean fluorescence intensity (MFI) as measured by flow cytometry. Bar graphs indicate median values with 95% confidence intervals (CI). Statistical differences between groups were calculated using a Mann-Whitney U test. **(E)** For correlative analyses between peak CD4$^+$ or CD8$^+$ CAR-T expansion levels and CD27 expression, a Pearson correlation coefficient was calculated across both patient groups. The black line shows the results of a linear regression analysis.

Supplemental Figure 2

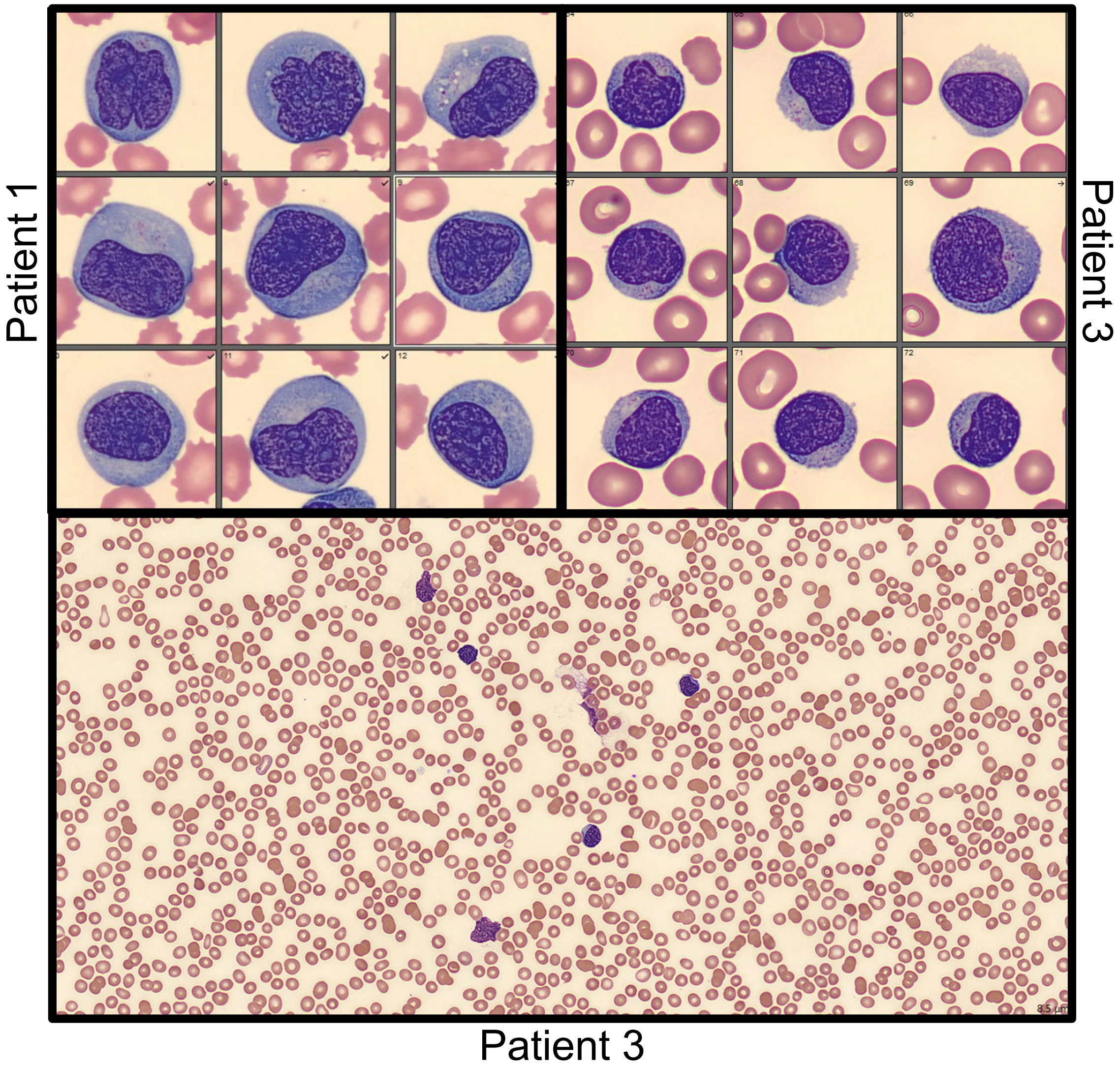

Supplemental Figure 2: Peripheral blood smears from patients receiving cilta-cel at the time of their CAR-T peak

Peripheral blood smears from two exemplary patients were stained by Wright Giemsa. The upper part shows atypical lymphocytes from the two patients, including large forms with ovoid to irregular nuclear contours, mature chromatin, and moderate amounts of palely basophilic cytoplasm. The lower half demonstrates a lower power magnification (200x) view of the peripheral blood smear from patient 3. These ”atypical” lymphocytes have repeatedly been confirmed to represent cilta-cel CAR T cells by flow cytometry.

Supplemental Figure 3

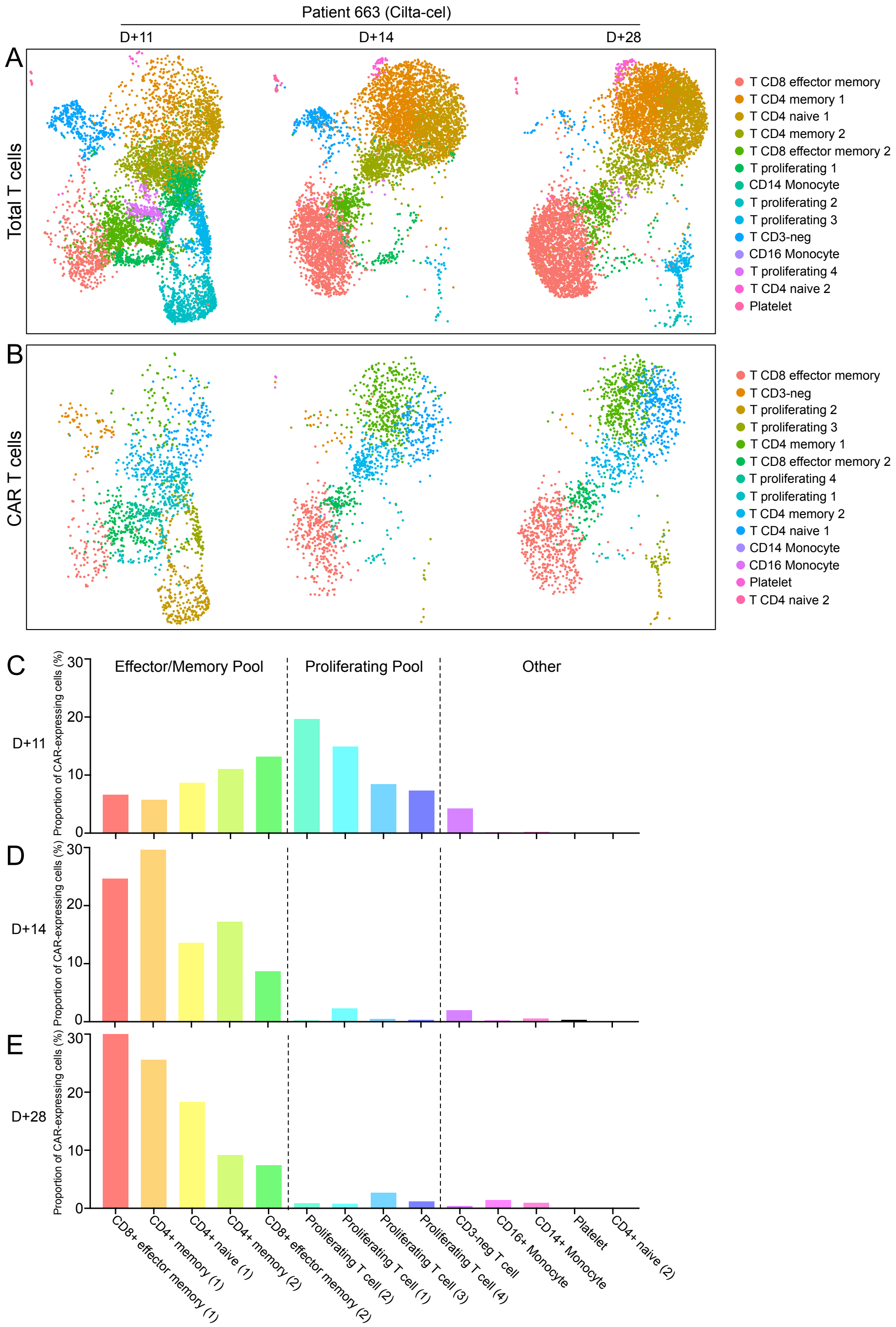

<u>Supplemental Figure 3:</u> **Rapid expansion and persistence of cilta-cel CAR T is associated with a change in gene signature over time**

To explore reasons for the strong CAR-T expansion in cilta-cel patients, we performed an analysis by single-cell RNA sequencing. We determined RNA expression patterns in serial blood samples from cilta-cel patient 663 from D+11, D+14, and D28 in **(A)** all cells combined and **(B)** in cells expressing the cilta-cel CAR. We were able to identify different subgroups of cells such as "effector/memory" and "proliferating" T cell pools among the CAR-T. Bar graphs indicate percentages of CAR-expressing cells belonging to the individual cell subtype within each group **(C-E)**.

Supplemental Figure 4

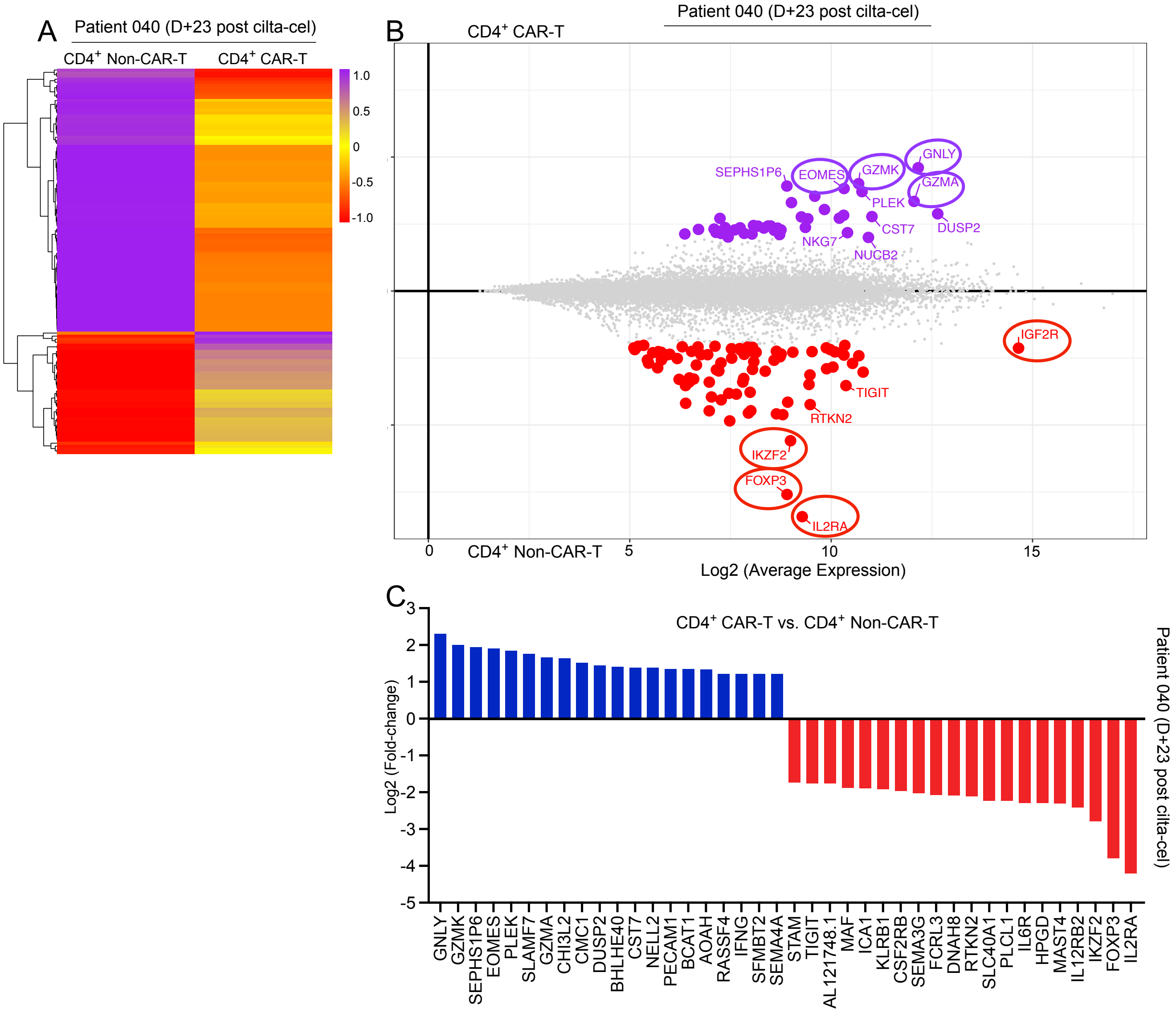

<u>Supplemental Figure 4:</u> RNA expression pattern in CAR-T vs. non-CAR-T from a cilta-cel patient

CD4+ non-CAR and CAR T cells were flow-sorted from a cilta-cel patient with strong CAR-T expansion at D+23 post CAR-T infusion and bulk RNA sequencing was performed. **(A)** Gene clustering of non-CAR and CAR T cells from the same patient and same timepoint. **(B)** M-A Plot illustrating the differentially expressed genes (DEGs) in the patient's CD4+ CAR T cells vs. non-CAR T cells. **(C)** Selection of the 40 genes most overexpressed (blue) or downregulated (red) genes in the CD4+ CAR T cells vs. non-CAR T cells. Bar graphs indicate Log2 fold-change.

Supplemental Figure 5

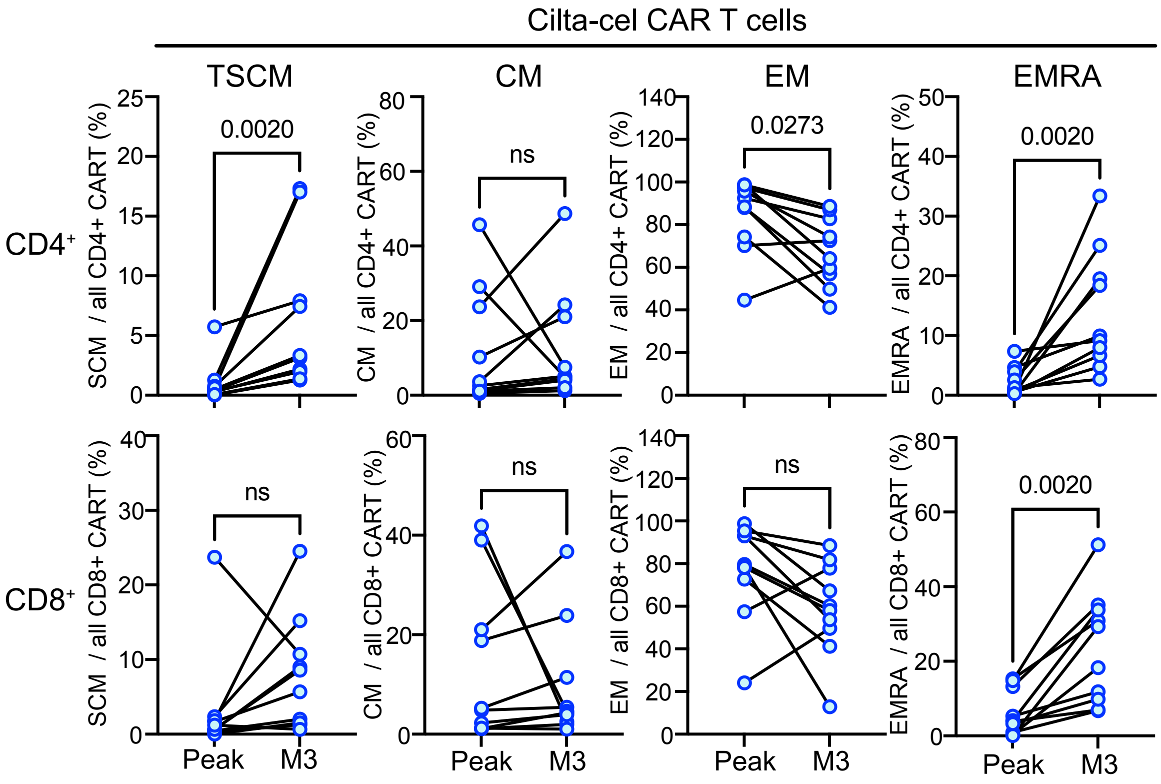

<u>Supplemental Figure 5:</u> Expansion of terminally differentiated effector CAR T cells in cilta-cel patients over time

Within the cilta-cel group, CD4⁺ and CD8⁺ CAR-T memory subsets (CM = of central memory; TSCM = T memory stem cells; EM = effector-memory; EMRA = terminal effector memory) were compared between the time of individual peak expansion and month three post CAR-T. For those patients where samples from both timepoints were available, memory subsets, as defined by expression of CD45RA and/or CD62L, were analyzed by flow cytometry. Timepoints within the same patient group were compared using a Wilcoxon test.

Supplemental Figure 6

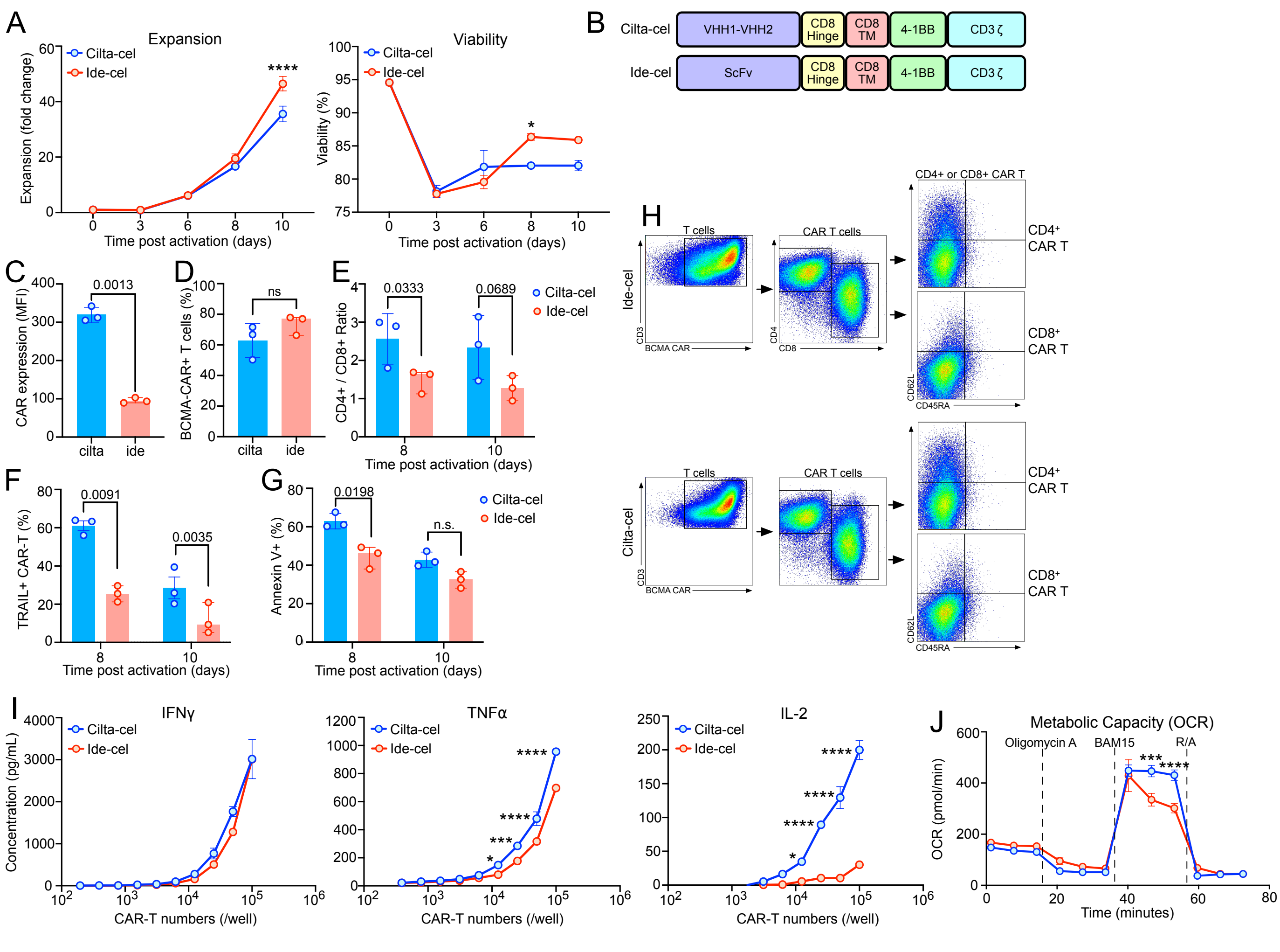

Supplemental Figure 6: The cilta-cel CAR T construct demonstrates greater metabolic capacity, tonic activation, and apoptosis *in vitro*.

(A) T cell expansion and viability of cilta-cel and ide-cel, which are **(B)** both comprised of a BCMA-targeting antigen binding domain (tandem VHHs domain or ScFv domain, respectively), followed by CD8 hinge and transmembrane domain, 4-1BB costimulatory domain, and CD3ζ costimulatory domain. were assessed at indicated times by trypan blue exclusion using Vi-CELL BLU. CAR surface expression in both products as indicated by **(C)** geometric mean fluorescence intensity (gMFI) or **(D)** percentage of CAR-expressing cells by flow cytometry. The **(E)** CD4/CD8 ratio among CAR+ T cells and the expression of apoptotic markers **(F)** TRAIL and **(G)** Annexin V were evaluated by flow cytometry after 8 and 10 days of *in vitro* culture. **(H)** The memory phenotype was assessed for both products on day 8 following costaining with CAR detection reagent and antibodies against different surface antigens followed by flow cytometry. **(I)** Spontaneous secretion of cytokines IFNγ, TNFα, and IL-2 from BCMA CAR T cell culture supernatants on day 9 was evaluated by ELISA. **(J)** BCMA-CAR mitochondrial metabolic capacity (oxygen consumption rate, OCR) of cilta-cel and Ide-cel was evaluated on day 10 with Seahorse XF T Cell Metabolic Profiling Kit using a Seahorse XFe96 Analyzer. Data are shown as mean ± SEM of experiments with CAR T cells generated from three healthy donors (A, C-G) or one representative donor of three tested in technical replicates (I+J). Statistical significance was determined by paired Student t-test or 2-Way ANOVA with Sidak's multiple comparisons; ns p>0.05; *p≤0.05; **p≤0.01; ***p≤0.001 ****p≤0.0001

Supplemental Figure 7

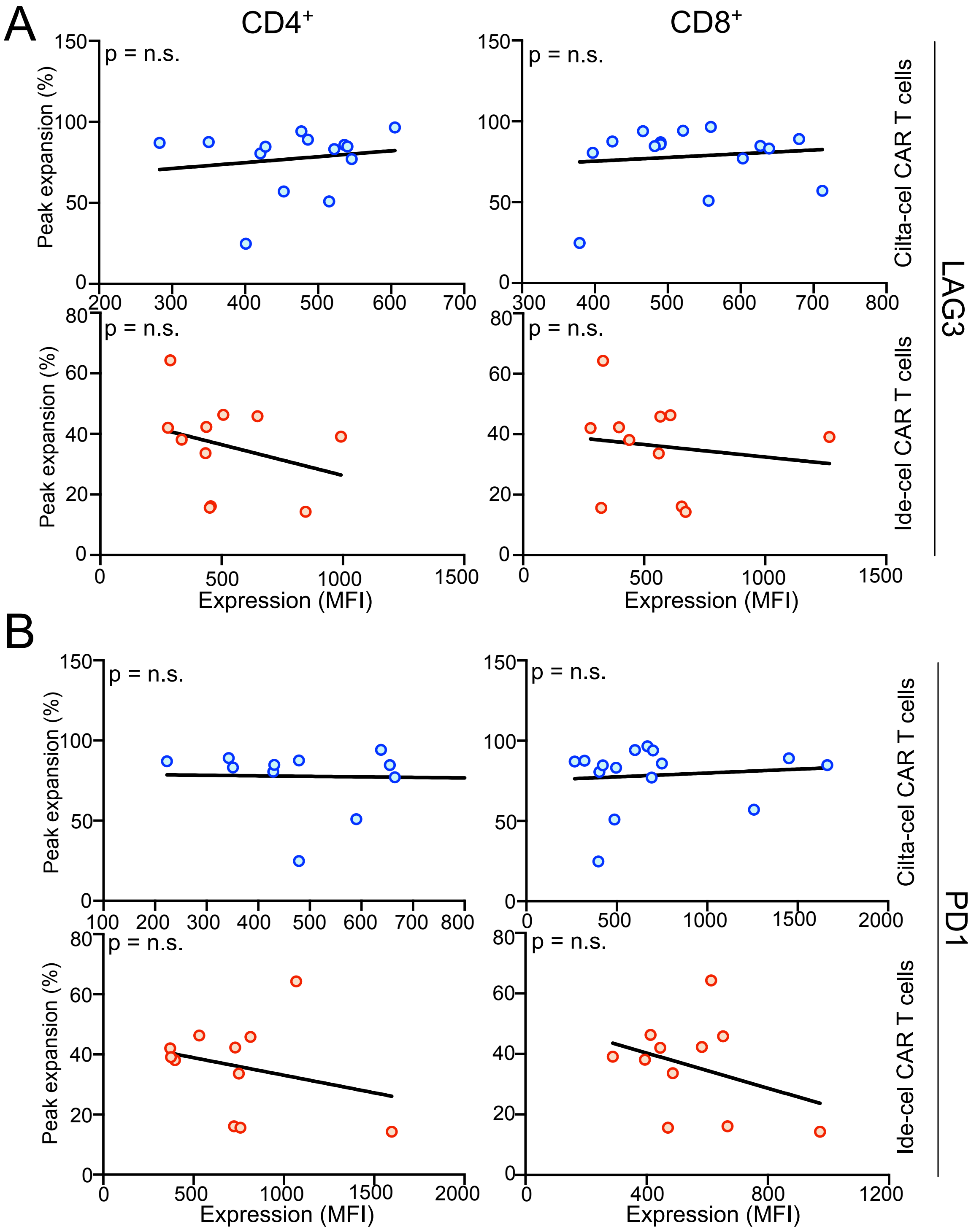

<u>Supplemental Figure 7:</u> Correlational analysis of the expression of exhaustion markers with CAR-T peak expansion

Levels of exhaustion markers LAG3 and PD1 were determined on the CAR-T in both patient groups. Dots show mean fluorescence intensity (MFI) of (A) LAG3 or (B) PD1 on $CD4^+$ and $CD8^+$ CAR-T at the time of peak expansion in both cilta-cel (blue) and ide-cel (red) patients. For correlative analyses between peak expansion levels and expression of a given exhaustion marker, a Pearson correlation coefficient was calculated.

Supplemental Figure 8

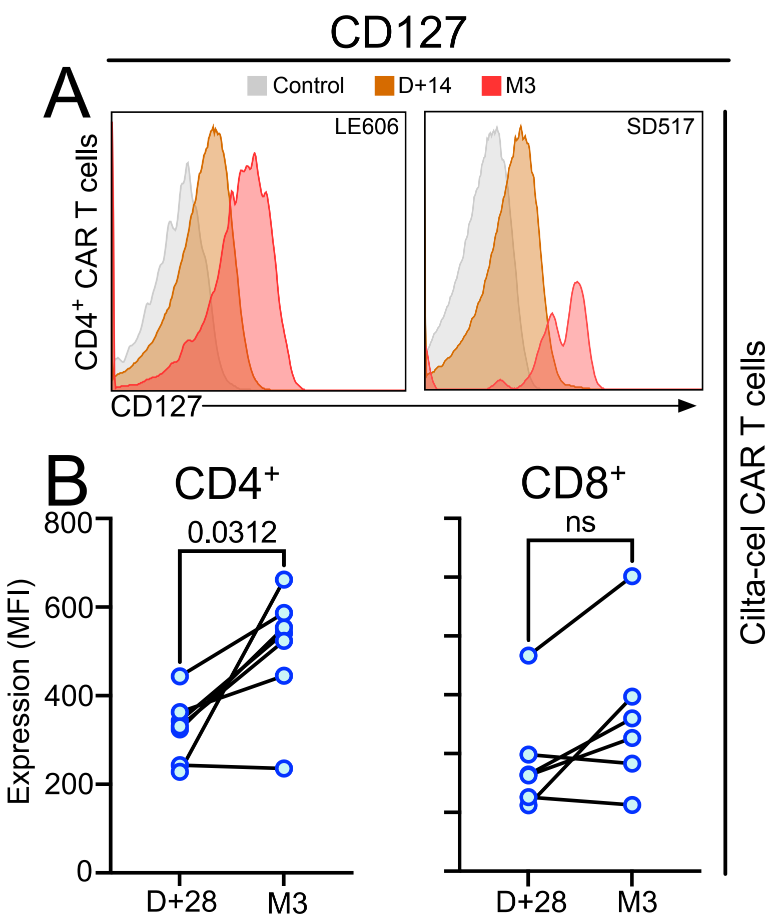

<u>Supplemental Figure 8:</u> CD127 is overexpressed on long-term persisting CAR-T from myeloma patients receiving cilta-cel

CD127 expression was measured on our patients' CAR-T over time by flow cytometry. The long-term persistent cilta-cel CAR-T had markedly upregulated expression of CD127 compared to earlier timepoints. **(A)** CD127 expression on two exemplary patients at D+14 and at three months (M3) after cilta-cel infusion. **(B)** Individual dots indicate mean fluorescence intensity (MFI) of CD127 expression on $CD4^+$ and $CD8^+$ CAR T cells at D+28 and at three months post CAR-T. Timepoints within the same patient group were compared using a Wilcoxon test.

Supplemental Figure 9

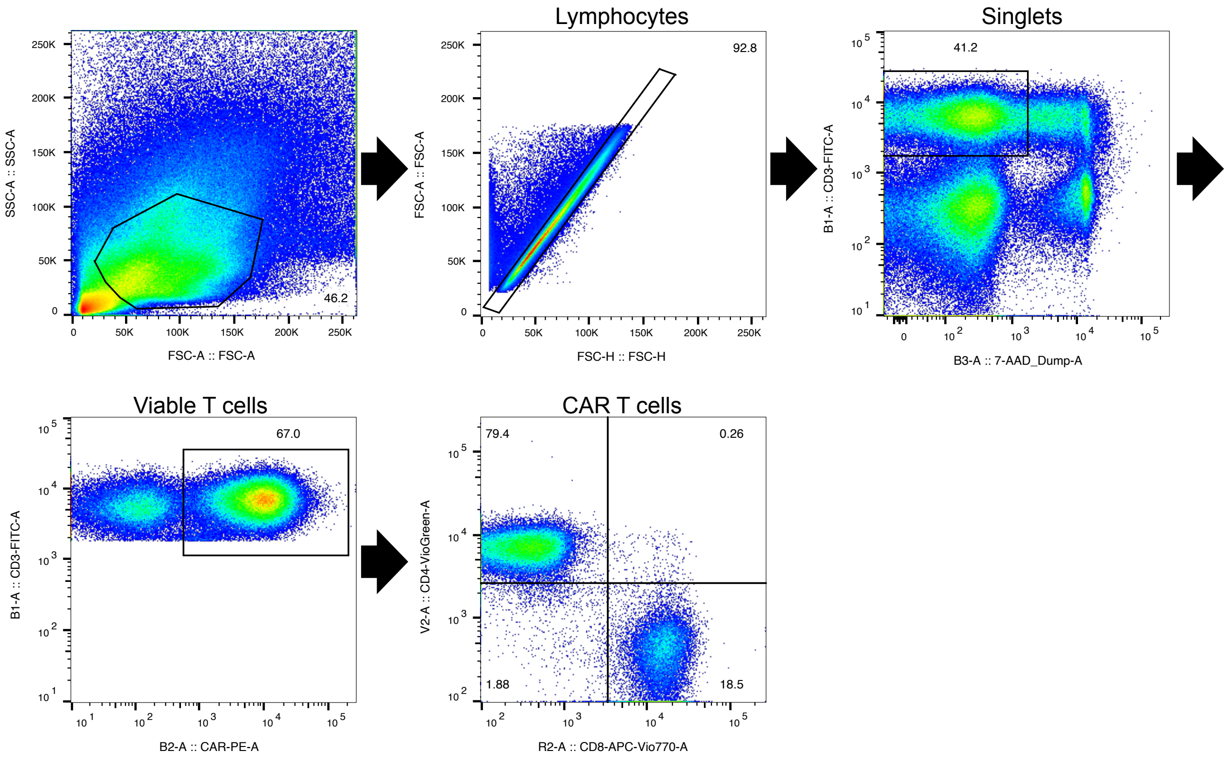

<u>Supplemental Figure 9:</u> Gating strategy for flow cytometry

The figure shows a general gating strategy applicable i.e. to Manuscript Figures 1, 2 B+C, 3E, 4 B-E, 5, and 6 A-C.

Supplemental Table 1: Monoclonal Antibodies for Flow Cytometry (CAR T Detection)

Target Antigen	Fluorophore	Manufacturer	CAT #
Biotin	PE	Miltenyi	130-110-951
CAR Detection (BCMA)	Biotin	Miltenyi	130-126-090
CAR Detection (CD19)	Biotin	Miltenyi	130-129-550
CD3	FITC	Miltenyi	130-113-138
CD19	PE-Vio770	Miltenyi	130-113-647
CD4	VioGreen	Miltenyi	130-113-230
CD8	APC-Vio770	Miltenyi	130-110-681
CD27	VioBlue	Miltenyi	130-120-036
CD45RA	VioBlue	Miltenyi	130-117-743
CD45RO	APC	Miltenyi	130-113-556
CD62L	PE-Vio770	Miltenyi	130-113-621
CD127	APC	Miltenyi	130-113-413
CD223 (LAG3)	VioBlue	Miltenyi	130-118-549
CD279 (PD1)	PE-Vio770	Miltenyi	130-120-385
CD366 (TIM3)	APC	Miltenyi	130-119-781

Supplemental Table 2: Reagents for Flow Cytometry (in vitro studies)

Target Antigen	Fluorophore	Manufacturer	CAT #
TRAIL	PE	Miltenyi	130-097-304
Annexin V	FITC	Miltenyi	130-093-060
CD4	VioBlue	Miltenyi	130-113-219
CD8	VioGreen	Miltenyi	130-113-164
7-AAD (Cell Viability solution)		BD Biosciences	555815
BCMA CAR Detection	Fc-conjugated	R&D Systems	193-BC
Fc (secondary antibody)	Alexa Fluor 647	Jackson ImmunoResearch	109-606-098

Supplemental Table 3: Types of neurotoxicities

Non-ICANS Neurotoxicity (Cilta-cel)	N (%)	
**Patients total**	**6**	**26.1**
Parkinson-like	3	13.0
Facial nerve paralysis	4	17.4
Transverse myelitis	1	4.3
RUE tremors	1	4.3

Supplemental Table 4: Types of infections

Post-CAR-T Infections (Cilta-cel)	N (%)	
**Patients total**	**18**	**78.3**
Bacterial	14	60.9
Viral	7	30.4
Fungal	1	4.3

SUPPLEMENTAL METHODS

CAR T cell treatment

All patients were treated using commercially available and approved CAR T cell products outside of clinical studies at the University of Maryland Greenebaum Comprehensive Cancer Center (UMGCCC). Aphereses in our cilta-cel patients took place between May 2022 and December 2023. CAR T cell products were administered following standard lymphodepleting chemotherapy with fludarabine and cyclophosphamide applied on three consecutive days. One patient had a bendamustine based regimen during the time of a fludarabine shortage in US. The fludarabine dose was dose adjusted as needed to the patient's renal function level.

Patient Samples

Samples and clinical data were collected under Institutional Review Board (IRB)-approved protocol 2043GCCC (IRB HP-00091736) for the immunomonitoring of lymphoma/myeloma patients following CAR-T treatment. In May of 2021 we opened the study at our institution and in April of 2024 we had enrolled 53 MM patients of whom 39 had at least one baseline sample and a minimum of 3 follow-up samples. Per protocol, blood samples were collected at apheresis, pre-lymphodepleting chemotherapy, day 0, day+7, day+14, day+21, day+28, month+3, and every 3 months thereafter. Additional unscheduled blood, bone marrow (BM), and cerebrospinal fluid (CSF) were collected if clinically indicated. Blood and BM samples were collected and plasma was generated by centrifugation at 400G and frozen immediately at -80°C. Peripheral blood mononuclear cells (PBMCs) were isolated using density gradient centrifugation and were either analyzed immediately or cryopreserved in liquid nitrogen until analysis. CSF samples were analyzed directly without additional prior processing.

Peripheral blood smears

Peripheral blood smears were generated as part of our routine follow-up by our Department of Hematopathology as described previously [1].

Flow Cytometry

For the *in vitro* experiments using CAR T cells generated in-house, cells were washed with staining buffer (autoMACS® Rinsing Solution with 0.5% BSA) and incubated with 0.2 μg of recombinant human BCMA-Fc Chimera Protein (R&D Systems, Minneapolis, MN) in the staining buffer (autoMACS® Rinsing Solution with 0.5% BSA) for 20 mins at 4℃. Then cells were washed twice and resuspended with staining buffer and incubated with anti-Fc-Alexa Fluor 647 at 1:200 dilution, antibodies of T cell surface markers, and 7-AAD for 15 mins at 4℃ (Supplemental Table 2). Cells labeled with surface markers were rinsed and resuspended in the Annexin V binding buffer and subsequently stained with Annexin V following the manufacturer's instructions (Miltenyi Biotec). Fluorescence Minus One (FMO) Controls were used in all the experiments to define CAR-positive T cell populations and they were also to assess expression of certain surface markers on the CAR T cells themselves such as CD27, CD127, TIM3, PD-1 and LAG-3.

RNA Sequencing

Bulk RNA Sequencing was performed by the Maryland Genomics core facility housed within the Institute for Genome Sciences at the University of Maryland Baltimore using cells sorted on a FACSAria flow cytometer (BD Biosciences, Franklin Lakes, NJ). Raw sequencing reads generated for each sample were analyzed using the CAVERN transcriptomics analysis pipeline [2]. Read quality was assessed using the FastQC toolkit to ensure quality reads for downstream analyses. Reads were aligned to the Human reference genome GRCh38 (available from Ensembl repository) using HISAT2, a fast splice-aware aligner for mapping next-generation sequencing reads [3]. Reads were aligned using default parameters to generate the alignment BAM files. Read alignments were assessed to compute gene expression counts for each gene using the HTSeq count tool [4] and the Human reference annotation (GRCh38). The raw read counts were normalized for library size and dispersion of gene expression. The normalized counts were utilized to assess differential gene expression between the CAR-T and non-CAR-T treated samples for

the same patient. 40 genes with the highest up-regulation and down-regulation were further utilized to assess the enrichment of gene ontology (GO) terms.

Single-cell RNA sequencing was performed by the Maryland Genomics core facility housed within the Institute for Genome Sciences at the University of Maryland Baltimore using 10x Genomics standard sequencing protocols. The sequencing reads were aligned to the human reference sequence (build GRCh38) and CAR sequence using CellRanger (10x Genomics, v6). The alignment outputs were used for preliminary quality control (QC), assessing the number of features detected, depth of gene expression (GE), and proportion of mitochondrial GE per sample. After QC, an integrated dataset was generated for all samples for each patient using the R package 'Seurat v4' [5] following the tutorial and vignette provided by the authors of Seurat. Principal Component Analysis followed by Uniform Manifold Approximation and Projection (UMAP) analysis was applied for cell clustering. Distinct cell clusters based on GE of highly variable genes were identified. GE patterns were assessed between clusters to find cluster-specific gene markers. Markers were compared to a publicly available database of cell type-specific genes using gene set enrichment analysis. Major cell types were identified, and cell clusters were assessed for known cell-type specific marker genes. Cell type proportions were assessed within each condition. Downstream analyses involved assessment of differentially expressed genes (DEGs) between conditions for each cell type. Significant differential expression was identified using an adjusted p-value cut-off of 0.05. All statistical analyses were performed, and illustrations were generated using R.

CAR-T cell generation for *in vitro* experiments

CAR T cells representing cilta-cel and Ide-cel were constructed based on publicly available sequences. DNA sequences were synthesized as gBlock and cloned into a lentiviral vector (LV) expression cassette under the control of the human EF-1α promoter (Lentigen Technology Inc., Gaithersburg, MD). Lentiviral vectors were generated by transient transfection using HEK 293T

cells, concentrated by centrifugation, and stored at -80℃. $CD4^+$ and $CD8^+$ T cells from apheresis material (HemaCare, CA or Miltenyi, MD) of healthy individuals were obtained from the supplier under informed consent. CAR-T transduction and culture were performed as previously described [6]. Briefly, positively selected $CD4^+$ and $CD8^+$ T cells were activated with MACS® GMP T Cell TransAct™ (Miltenyi Biotec) at a density of $1x10^6$ cells/ml with 30 IU/ml of recombinant human IL-2 (Miltenyi Biotec) in the TexMACS™ GMP Medium (Miltenyi Biotec) at day 0. T cells were then transduced with CAR-expressing LV at MOI 20 the next day. On day 3, the T cells were washed and resuspended in fresh TexMACS media with IL-2. The T cells were expanded until day 10.

Enzyme-linked immunosorbent assay (ELISA)

Supernatants harvested from CAR T-cell cultures in the absence of target cells, plated at the indicated cell number per 96-well in 200 ul TexMACS medium without supplements or cytokines, were analyzed for IFN-γ, TNF-α, and IL-2 concentration by ELISA (ThermoFisher Scientific, Inc., Waltham, MA) following the manufacturer's instructions.

Metabolic Profiling

The assay was performed using the Seahorse XF T Cell Metabolic Profiling Kit (Agilent, Santa Clara, CA) following the manufacturer's instructions. Briefly, the sensor cartridge and PDL cell culture plate were prepared on one day before the assay. On the day of the assay, T cells were suspended in the Seahorse XF RPMI medium with glucose (10 mM), pyruvate (1 mM), and glutamine (2 mM) and plated in pre-warmed Seahorse XF PDL cell culture plate. The plate was then incubated at 37℃ without CO2 for 45 to 60 mins. The three compounds with indicated final concentrations, Oligomycin (1.5 μM), BAM15 (2.5 μM), and Rotenone/Antimycin A (0.5 μM each), were subsequently loaded into injection ports. Oxygen consumption rate (OCR) was assessed by Seahorse XFe96 analyzer (Agilent).

References for Supplemental Methods

1. Kallen, M.E., R. Koka, and D. Atanackovic, *Carvykti CAR T-cell morphology in cellavision peripheral smear reviews.* Journal of Hematopathology, 2024.
2. Shetty, A., et al. *CAVERN: Computational and visualization environment for RNA-seq analyses*. in *Proceedings of the 69th Annual Meeting American Society of Human Genetics*. 2019.
3. Kim, D., B. Langmead, and S.L. Salzberg, *HISAT: a fast spliced aligner with low memory requirements.* Nature Methods, 2015. **12**(4): p. 357-360.
4. Anders, S., P.T. Pyl, and W. Huber, *HTSeq—a Python framework to work with high-throughput sequencing data.* Bioinformatics, 2014. **31**(2): p. 166-169.
5. Hao, Y., et al., *Integrated analysis of multimodal single-cell data.* Cell, 2021. **184**(13): p. 3573-3587.e29.
6. Tran, T.M., et al., *Armored TGFbetaRIIDN ROR1-CAR T cells reject solid tumors and resist suppression by constitutively-expressed and treatment-induced TGFbeta1.* J Immunother Cancer, 2024. **12**(4).
